# Evaluation of an Enhanced Sleep Education Programme in Promoting Sleep and Circadian Health in Adolescents

**DOI:** 10.1192/j.eurpsy.2025.507

**Published:** 2025-08-26

**Authors:** A. H. Pang, W. Y. Chan, H. F. Sit, S. X. Li, F. T. W. Cheung

**Affiliations:** 1 Sleep Research Clinic and Laboratory, Department of Psychology; 2 School of Public Health, LKS Faculty of Medicine, The University of Hong Kong; 3 Department of Psychology, The Education University of Hong Kong; 4 The State Key Laboratory of Brain and Cognitive Sciences, The University of Hong Kong, Hong Kong, China

## Abstract

**Introduction:**

Sleep problems, particularly insomnia and sleep deprivation, are common among adolescents, and may increase their risk for poor psychosocial and metabolic health. Traditional classroom-based education programmes showed inconsistent results in promoting sleep behavioural changes. In contrast, enhancing training programme with experiential activities alongside classroom learning may be considered as a more sustainable means to promote healthy sleep in adolescents.

**Objectives:**

The present study evaluated the impact of a four-week enhanced sleep education programme on adolescents’ sleep quality, knowledge and behaviour, and their experience of participation.

**Methods:**

Adolescent participants took part in the Youth Sleep Ambassadors Programme, which was designed to equip them with evidence-based information on sleep and circadian health through active learning and community outreach activities. A mixed-methods design was adopted to evaluate the programme. Participants completed self-administered questionnaires, including a sleep knowledge quiz, the Pittsburgh Sleep Quality Index (PSQI) to assess subjective sleep quality, and the Sleep Hygiene Index (SHI) to evaluate healthy sleep behaviour, before and after the programme. Additionally, two focus groups were conducted to understand participants’ experiences with the programme.

**Results:**

Thirteen participants, aged 15 to 17, participated in the progamme and rated the programme’s effectiveness at 4.38 out of 5. At post-programme, there were significant increases in total sleep time (baseline: 6.12hr, post: 7.31hr, *p*=.006) and time in bed (baseline: 6.83hr, post: 7.98hr, *p*=.046), in addition to a reduction in PSQI score (baseline: 5.92±1.98, post: 3.92±1.50, *p*=.031). There was a noticeable trend of improved sleep knowledge with a 13.5% increase in correct rate and healthy sleep behaviour (SHI: baseline: 24.4±6.76, post: 20.6±5.33). Focus groups revealed increased awareness and confidence to advocate for better sleep and mental health among peers. Participants praised the programme as professional and well-organised but expressed a preference for more experiential and research activity over classroom learning, underscoring the limitations of classroom-based sleep education.

**Conclusions:**

The findings provided preliminary support for this enhanced sleep education programme as a viable strategy to engage adolescents in understanding and awareness of sleep and circadian health. Beyond traditional classroom education, self-discovery and knowledge application with experiential tasks can better develop their perspectives and advocate in their community.

**Disclosure of Interest:**

None Declared

